# Health-related quality of life in T1DM patients after high-dose cholecalciferol supplementation: data from a pilot clinical trial

**DOI:** 10.1186/s13098-022-00817-w

**Published:** 2022-03-28

**Authors:** Ana Carolina Contente Braga de Souza, Maria Clara Neres Iunes de Oliveira, Gabriela Nascimento de Lemos, Emanuele Rocha da Silva, Ícaro José Araújo de Souza, Wanderson Maia da Silva, Angélica Leite de Alcântara, Nivin Mazen Said, Lorena Vilhena de Moraes, João Felício Abrahão Neto, Simone Rodrigues dos Passos, Ádria Aline Alves Monteiro, Natércia Neves Marques de Queiroz, Franciane Trindade Cunha de Melo, Karem Miléo Felício, Lilian de Souza D’Albuquerque Silva, Daniela Lopes Gomes, Neyla Arroyo Lara Mourão, Pedro Paulo Freire Piani, Isabel Jane Campos Lobato, João Soares Felício

**Affiliations:** Endocrinology Division, University Hospital João de Barros Barreto, Federal University of Pará, Mundurucus Street, 4487, Guamá, Belém, Pará 66073-000 Brazil

**Keywords:** Type 1 diabetes mellitus, Quality of life, Vitamin D, Albuminuria

## Abstract

**Background:**

Type 1 Diabetes Mellitus (T1DM) impacts health-related quality of life (HRQoL). Cross-sectional studies suggest that low levels of vitamin D (VD) may impair HRQoL, however, the effect of VD supplementation on quality of life in T1DM patients has not yet been clarified. Our study evaluated the effects of high-dose VD supplementation on HRQoL in T1DM.

**Methods:**

We performed a prospective study with 64 patients receiving cholecalciferol (4000 IU/day for patients with 25-OH-vitamin D [25(OH)D] between 30 and 60 ng/mL, and 10,000 IU/day for those with 25(OH)D below 30 ng/mL) for 12 weeks, as part of a research protocol. HRQoL was assessed with EuroQol instruments (EQ-5D and EQ-VAS).

**Results:**

There was an improvement in global EQ-5D index, and analysing specifically the EQ-5D domains, we observed an improvement in mobility (1.3 ± 0.6 versus 1.1 ± 0.3, p < 0.01). Evaluating possible outcome influencing variables, we detected a reduction in albuminuria at the end of the trial, without changes in BMI, lipids, blood pressure, glycemic control and insulin doses. We found correlations between final albuminuria and the dimensions: mobility (r = 0.6; p < 0.01), personal care (r = 0.7; p < 0.01), pain and discomfort (r = 0.6; p < 0.01) and habitual activities (r = 0.6; p < 0.01), suggesting an association between albuminuria reduction and the impact of VD supplementation on HRQoL.

**Conclusion:**

Our data showed that high doses of cholecalciferol supplementation can improve HRQoL in patients with T1DM, and the reduction of albuminuria seems to be an important factor in this context.

*Trial registration*: (ISRCTN32601947), 03/06/2017 retrospectively registered.

## Background

Type 1 diabetes mellitus (T1DM) is the most common endocrinopathy of childhood and adolescence and it is characterized by severe insulin deficiency, corresponding to approximately 10% of all diabetes cases, involving approximately 10 to 20 million individuals worldwide [1]. Due to its high complexity, it is a disease with important physical, psychological and social repercussions and a considerable impact on the quality of life (QoL) of patients and their families [2].

The Brazilian Type 1 Diabetes Study Group (BrazDiab1SG) evaluating over 3000 patients with T1DM, collected information from demographic, economic, clinical-laboratory and health-related quality of life (HRQoL), which was measured using EuroQol [3]. Those data showed that variables as HbA1c, age, socioeconomic status (SES), physical activity, and the presence of micro and macrovascular complications were weakly associated with QoL, suggesting the need to investigate other factors that may affect this scenario [3].

Vitamin D levels are lower in DM patients [4, 5], but whether hypovitaminosis D is associated or not with impared quality of life and if cholecalciferol high-dose supplementation could improve this scenario in this group remains controversial [6–9]. Thus, the aim of our study was to evaluate the effect of high-dose cholecalciferol supplementation on HRQoL of patients with T1DM.

## Methods

### Study design and patients

We performed a pilot prospective study in order to evaluate the effect of high dose vitamin D supplementation in health-related quality of life (HRQoL) in patients with T1DM as part of a research protocol (ISRCTN32601947) that has already provided evidence on other aspects of VD supplementation outcomes [10–13]. This trial was reviewed and approved by University Hospital João de Barros Barreto (HUJBB) ethics committee.

A total of 64 subjects were recruited from the endocrinology ambulatory department from HUJBB, signed consent was obtained from all patients. Participants were divided in two groups: patients with basal VD levels between 30 and 60 ng/mL (who received 4.000 IU /day of cholecalciferol) and patients with basal VD levels below 30 ng/mL (supplemented with 10,000 IU/day), both for 12 weeks. Our intention was to asegurate that patients achieve and maintain VD levels between 30 and 60 at the end of the trial to maximize VD extra-skeletal actions, without collateral effects. Because of that, we used the dose ranged between 4.000 and 10,000 IU/day according to the recommendation of Endocrine Society [14].

Inclusion criteria consisted in: (a) patient with T1DM diagnosis aged between 12 and 50 years, in at least a 1-year follow-up and in regular treatment with an endocrinologist; (b) glycated hemoglobin ≥ 7%; (c) insulin dose stability for at least 3 months before participating in the study; (e) NPH, Glargine, Detemir, Aspart, Glulisin, Lispro, and Regular insulin were allowed; (f) patients in use of metformin could participate of the study as long as they were using the same dose for at least 3 months; (g) patients with hypertension and/or diabetic kidney disease (DKD) must be in stable doses of antihypertensive medication for at least four weeks; (h) compliance with diet and exercise regimen. Exclusion criteria were: (a) history of hepatic or bone metabolism disorders; (b) previous VD or Calcium supplementation; (c) abnormal serum creatinine levels (d) anemias; (e) breastfeeding and pregnant women; (f) uncontrolled hypo or hyperthyroidism and allergies to VD supplementation; (g) hemotransfusion and/or blood donation within the 3 months before the first visit. Patients were previously instructed to maintain physical activity regularly according to American Diabetes Association Guidelines [15] to avoid variations during the study.

### Data collection

Data collection occurred during scheduled visits, during pre-treatment (baseline) and post-treatment (end of study) phases. Analysis of medical records (demographics, pre-existent clinical conditions, insulin and other medications in use) and physical examination were carried out. Laboratory tests and health-related quality of life evaluation (EQ-5D-5L and EQ- VAS) [16] were performed before and after twelve weeks.

Serum 25(OH)D was measured by DiaSorin LIAISON 25-OH-Vitamin D TOTAL chemiluminescence immunoassay (DiaSorin, Stillwater, MN, USA) [17, 18]; HbA1C was analyzed by HPLC method. Fasting glucose, triglycerides, total cholesterol, low-density lipoprotein cholesterol (LDL-C) and high-density lipoprotein cholesterol (HDL-C) were measured by colorimetry. The ultra-sensitive C-reactive protein was evaluated by ARCHITECT turbidimetry, and creatinine by the kinetic/automated method. The glomerular filtration rate **(**GFR) was calculated by the CKD-EPI formula [19]. Albuminuria was measured in three 24 h urine samples by immunoturbidimetry [20]. Peripheral neuropathy was evaluated by two scores: Total Symptoms Score (TSS) and Neuropathy Disability Score (NDS). Presence of this complication was established by TSS >  = 2 and NDS >  = 3 [21, 22].

### Quality of life assessment

Quality of life was assessed by the EuroQol 5 and its two tools: the EQ-5D-5L and EQ-VAS [16]. The first one descriptively analyzes five dimensions of problems: mobility, self-care, pain and discomfort, usual activities, and anxiety and depression. Each dimension has five levels from "no problems" to "extreme problems". The EQ-VAS consists of an analog scale from 0 (poor health status) to 100 (optimal health status) for the patient to mark/say a value that reflects, in his/her perception, his/her health status. The HRQoL assessment tool was applied at basal and final visits.

The health states defined by the EQ-5D-5L responses were translated into EQ-5D utility index by means of sets of values that were derived from large population-based surveys. The utility index scale ranges from 0.0 to 1.0, where 0.0 represents death and 1.0 represents full health. As we did not have a publicly available set of EQ-5D-5L values for Brazilian population, in our study we used the set of values from the following countries: United States, Spain, Zimbabwe, Denmark, France, Germany, Japan, Netherlands, Thailand, and United Kingdom using the link (https://euroqol.org/eq-5dinstruments/eq-5d-5l-about/valuation-standard-value-sets/crosswalk-index-value-calculator/).

### Statistical analysis

Before and after VD supplementation, categorical variables were compared by Chi-square, Fisher and McNemar tests, while numerical ones were analysed with Student's T and Mann–Whitney tests, with and without normal distribution, respectively. Also, Paired Student's T and Wilcoxon tests were used to compare paired groups before and after the follow-up period, with normal and non-normal distribution, respectively. Data with normal distribution were represented as mean and standard deviation values, while data with non-normal distribution were represented by median and quartiles. To compare more than two groups, the analysis of variance test (ANOVA) was applied for normal distribution variables and Kruskal–Wallis test for non-normal distribution ones. To calculate the sample size, we used paired T test. We expected a change to be detected = 0.2, with an expected standard deviation exchange of 0.5 with a desired power = 0.8 in EQ-5D-5L dimensions. The sample size to achieve it was 52. Additionally, albuminuria values were converted to log base 10 (log10) to better analyze the data. The level of statistical significance was set at p < 0.05 and all analyses were stored and processed with the software SigmaStat (Jandel Scientific) version 3.5 and SPSS (Statistical Package for Social Sciences) 22.0 IBM.

## Results

Clinical characteristics at the beginning of the study are presented in Table 1.

**Table 1:** Clinical characteristics at the beginning of the study

Clinical characteristics	N = 64
Age (Years)	27.6 ± 10.1
Gender (F/M)	33/31
DM duration (Years)	12.1 ± 8.1
History of dyslipidemia (Yes %)	15 (23.4%)
History of hypertension (Yes %)	10 (15.6%)
History of DKD (Yes %)	20 (31.2%)
History of Diabetic Retinopathy (Yes %)	11 (17.1%)
Smoking (Yes %)	10 (15.6%)
Previous treatment with ACEI/ARB (Yes %)	18 (28.1%)

*T1DM* type 1 diabetes mellitus, *DKD* diabetes kidney disease, *ACEI* angiotensin-converting enzyme inhibitors, *ARB* angiotensin II receptor blockers, *F* female, *M* male

Most patients of our sample (72%) had 25(OH)D levels below 30 ng/ml, and, by the end of the trial, 94% of T1DM patients were classified with normal VD (≥ 30 ng/ml) (p < 0.001).

In Table 2 are described the main clinical and laboratorial parameters before and after VD supplementation. By the end of the study, most analyzed parameters have not changed, except for 25(OH)VD levels (26.7 ± 9 versus 55.1 ± 24.1 ng/mL, p < 0.001).

**Table 2:** Clinical and laboratory parameters of patients with T1DM before and after vitamin D supplementation

Parameters (N = 64)	Pre VD	Post VD	P
BMI (kg/m^2^)	24 ± 4	24 ± 4	NS (0.247)
Systolic Blood Pressure (mmHg)	115 ± 11	115 ± 12	NS (0.765)
Diastolic Blood Pressure (mmHg)	71 ± 9	72 ± 9	NS (0.153)
Basal insulin dose (IU)	35.6 ± 17	36 ± 18	NS (0.957)
Prandial insulin dose (IU)	22.3 ± 12	23.3 ± 12	NS (0.177)
Total insulin dose (IU)	55.8 ± 27	58.1 ± 27	NS (0.708)
25(OH)VD (ng/mL)	26.7 ± 9	55.1 ± 24	< 0.001
HbA1c (%)	9.6 ± 2	9.8 ± 3	NS (0.250)
Fasting plasma glucose (mg/dL)	168 ± 96	180.6 ± 103	NS (0.497)
Ultrasensible CRP (mg/L)	0.36 ± 0.52	0.37 ± 0.54	NS (0.727)
Total cholesterol (mg/dL)	171.7 ± 41	177 ± 54	NS (0.292)
HDL cholesterol (mg/dL)	52 ± 38	44.6 ± 11	NS (0.293)
LDL cholesterol (mg/dL)	103.7 ± 31	107.9 ± 49	NS (0.714)
Non-HDL cholesterol (mg/dL)	123.5 ± 38	125.7 ± 50	NS (0.289)
Triglycerides (mg/dL)	97.7 ± 51	109.6 ± 70	NS (0.186)

*IU* international units, *NS* non significant, *BMI* body-mass index, *HbA1c* glycated hemoglobin, *CRP* c-reactive protein, *GFR* glomerular filtration rate, *HDL* high-density lipoprotein, *LDL* low-density lipoprotein

In Table 3 we described EQ-5D utility index in all countries with available datasets given by EuroQol. It included countries that had common geographic conditions as that of Brazil (Thailand and Zimbabwe) or not (Denmark and Japan). We used all EuroQol datasets available. The results were consistently the same. On HRQoL evaluation by EuroQol, we found an improvement on EQ-5D-5L utility index by the end of the study, according to electronic calculators available from evaluated countries, independently of Human Development Index (HDI) (Table 3).

**Table 3:** Utility index of EQ-5D before and after vitamin D supplementation using a set of values of available countries

Country	HDI	EQ-5D index before VD	EQ-5D index after VD	P
USA	0.926	0.87 ± 0.1	0.90 ± 0.1	< 0.05
Spain	0.904	0.88 ± 0.1	0.91 ± 0.1	< 0.05
Zimbabwe	0.571	0.83 ± 0.1	0.85 ± 0.1	< 0.05
Denmark	0.940	0.86 ± 0.1	0.89 ± 0.1	< 0.05
France	0.901	0.84 ± 0.2	0.89 ± 0.2	< 0.05
Germany	0.947	0.92 ± 0.1	0.94 ± 0.1	< 0.05
Japan	0.919	0.83 ± 0.1	0.87 ± 0.1	< 0.05
Thailand	0.777	0.79 ± 0.2	0.85 ± 0.2	< 0.01
UK	0.932	0.84 ± 0.2	0.88 ± 0.2	< 0.05

*HDI* human development Index, *UK* United Kingdom, *VD* Vitamin D

In EQ-5D, we observed an important improvement in mobility (1.3 ± 0.6 versus 1.1 ± 0.3, p < 0.01) and there was also a tendency to improve in self-care dimension at the end of VD supplementation. We did not find additional changes in the other EQ-5D dimensions, as in the attributed grade to general health status, evaluated by EQ-VAS (Table 4)

**Table 4:** Analysis of EuroQol dimensions and grade of EQ-VAS before and after vitamin D supplementation

EQ-5D-5L dimensions (N = 64)	Before VD	After VD	p
1. Mobility	1.3 ± 0.6	1.1 ± 0.3	< 0.01
2. Self-care	1.1 ± 0.4	1.0 ± 0.3	0.125
3. Usual activities	1.3 ± 0.6	1.5 ± 0.7	0.669
4. Pain and discomfort	1.6 ± 0.8	1.5 ± 0.7	0.261
5.Anxiety and Depression	1.4 ± 0.7	1.4 ± 0.8	0.847

EQ-VAS	Before VD	After VD	P
Referred value	81 ± 17	80 ± 20	0.913

*VD* Vitamin D

Main DM chronic complications before and after VD administration are shown on Table 5. We observed significant reduction in albuminuria (1.27 ± 0.7 versus 1.22 ± 0.7, p = 0.01), with no changes in the other parameters.

**Table 5:** Clinical and laboratorial data concerning chronic T1DM complications before and after vitamin D supplementation

Parameters (N = 64)	Pre VD	Post VD	P
Periferic neuropathy (Yes %)	25%	17%	NS (0.131)
PN – TSS	1.8 ± 2.3	1.6 ± 2.5	NS (0.166)
PN – NDS	2.1 ± 2.5	1.8 ± 2.6	NS (0.262)
Albuminuria (mg/24 h – Log10)	1.27 ± 0.7	1.22 ± 0.7	0.01
GFR (ml/min/1.73m2)	120 ± 37	117 ± 36	NS (0.247)

*NS* non significant, *GFR* glomerular filtration rate, *HDL* high-density lipoprotein, *LDL* low Density lipoprotein, *PN* peripheral neuropathy, *TSS* total symptoms score—neuropathy, *NDS* neuropathy disability score

**Fig. 1:**
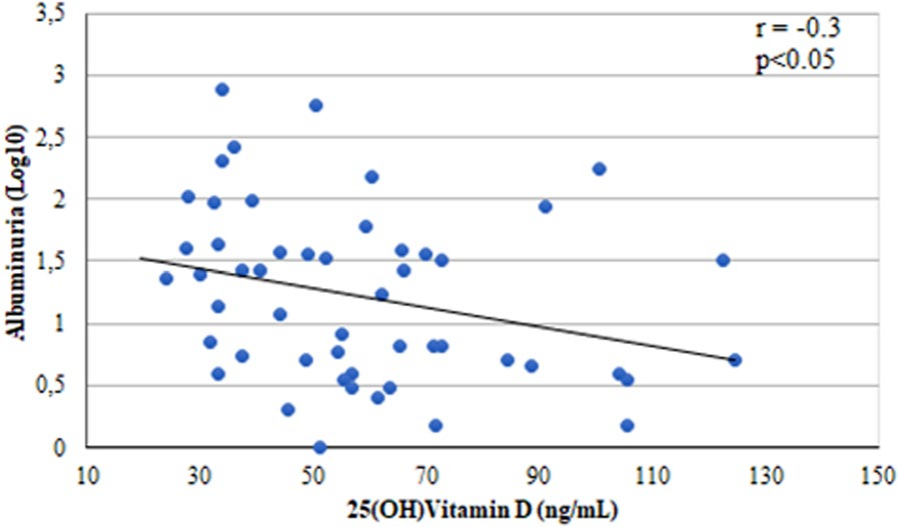
Correlation between albuminuria and 25(OH)D levels at the end of the study

Additionally, it was observed that final 25(OH)D levels showed correlation with final albuminuria (r = − 0.3, p < 0.05) (Fig. 1). Therefore, the higher the 25(OH)D levels, the lower the albuminuria excretion rates by the end of VD supplementation.

In a post-hoc analysis, we compared patients who improved (group 1A, N = 54) or not (group 1B, N = 10) the EQ-5D utility index. We found an albuminuria reduction at the end of VD administration only in group 1A. No other differences were identified, except for increase in non-HDL and LDL cholesterol levels in group 1B at the end of the study (Table 6).

**Table 6:** Analysis of clinical and laboratorial parameters according to improvement (group 1A) or not (group 1B) of the EQ-5D utility index after vitamin D supplementation

	Group 1A (N = 54)		Group 1B (N = 10)		
Parameters	Pre VD	Post VD	Pre VD	Post VD	p
BMI (kg/m^2^)	24 ± 4	24 ± 4	22 ± 3	22 ± 2	NS
Peripheral neuropathy yes/no (%)	(13/54) 25%	(8/54) 16%	(1/10) 10%	(1/10) 10%	NS
Basal insulin dose (IU)	36 ± 18	37 ± 19	33 ± 18	30 ± 14	NS
Prandial insulin dose (IU)	23 ± 11	24 ± 11	20 ± 13	21 ± 12	NS
25(OH)VD (ng/mL)	27 ± 9	57 ± 25	26 ± 8	52 ± 23	< 0.001^a,b^
HbA1c (%)	9.5 ± 2.4	9.6 ± 2.5	10.1 ± 2.4	10.6 ± 3.6	NS
HDL cholesterol (mg/dL)	47 ± 12	45 ± 10	50 ± 11	49 ± 13	NS
LDL cholesterol (mg/dL)	105 ± 33	105 ± 51	97 ± 19	122 ± 35	< 0.005^b,*^
Non-HDL cholesterol (mg/dL)	123 ± 41	122 ± 52	117 ± 23	147 ± 45	0.01^b^
Triglycerides (mg/ dL)	97 ± 53	108 ± 75	101 ± 43	111 ± 53	NS
Albuminuria (mg/24 h – Log10)	1.25 ± 0.75	1.2 ± 0.75	1.27 ± 0.68	1.24 ± 0.4	< 0.05^a^

*NS* non significant, *BMI* body-mass index, *HbA1c* glycated hemoglobin, *CRP* c-reactive protein, *GFR* glomerular filtration rate, *HDL* high-density lipoprotein, *LDL* low-density lipoprotein

^*^Group 1A differs from group 1B after VD

^a^Group 1A differs before and after VD

^b^Group 1B differs before and after VD

Furthermore, we performed a second post-hoc analysis on patients with improvement (group 2A, n = 25) or not EQ-VAS (group 2B, n = 39), and found a significant increase in the dose of prandial insulin in group 2B (22.1 ± 13.1 IU versus 24.4 ± 12 IU, p < 0.05). Albuminuria reduced significantly only in group 2A (1.2 ± 0.7 versus 0.98 ± 0.7; p < 0.05). Besides that, we found that final albuminuria excretion was significantly lower in group 2A when compared to group 2B (Table 7).

**Table 7:** Analysis of clinical and laboratorial parameters according to improvement (Group 2A) or not (Group 2B) of EQ-VAS at the end of vitamin D supplementation

	Group 2A (N = 25)		Group 2B (N = 39)		
Parameters	Pre VD	Post VD	Pre VD	Post VD	p
BMI (kg/m^2^)	23.3 ± 4	23.4 ± 4	24.3 ± 5	24.3 ± 5	NS
Peripheral neuropathy yes/no (%)	(4/21) 16%	(3/22) 12%	(12/27) 30%	(8/31) 21%	NS
Basal insulin dose (IU)	36 ± 14.2	39 ± 19	35 ± 19	34 ± 19	NS
Prandial insulin dose (IU)	22.7 ± 9	21.7 ± 10	22.1 ± 13	24.4 ± 12	< 0.05^a^
25(OH)VD (ng/mL)	26.1 ± 9	62.7 ± 27	27.1 ± 9	50.3 ± 20	< 0.001^a,b^
HbA1c (%)	9.6 ± 2	9.7 ± 3	9.6 ± 2	9.8 ± 3	NS
HDL cholesterol (mg/dL)	47 ± 10	44 ± 8	48 ± 13	46 ± 12	NS
LDL cholesterol (mg/dL)	126 ± 27	122 ± 30	122 ± 43	128 ± 58	NS
Non-HDL cholesterol (mg/dL)	88 ± 28	96 ± 46	107 ± 60	118 ± 83	NS
Albuminúria (mg/24 h – Log10)	1.2 ± 0.7	0.98 ± 0.7	1.3 ± 0.8	1.4 ± 0.7	< 0.05^b*^

*NS* non significant, *BMI* body-mass index, *HbA1c* glycated hemoglobin, *CRP* c-reactive protein, *GFR* glomerular filtration rate, *HDL* high-density lipoprotein, *LDL* low-density lipoprotein

^*^Group 2A differs from Group 2B after VD

^a^Group 2B differs before and after VD

^b^Group 2A differs before and after VD

On patients with an improvement of EQ-VAS at the end of the study (Group 2A), we also observed a correlation between final albuminuria and the following dimensions of EQ-5D: mobility (r = 0.6; p < 0.01), self-care (r = 0.7; p < 0.01), habitual activities (r = 0.6; p < 0.01) and pain and discomfort (r = 0.6; p < 0.01).

## Discussion

Our findings suggested an improvement in T1DM patients quality of life after supplementation with high doses of cholecalciferol. According to our knowledge, this is the first clinical trial to demonstrate the impact of VD supplementation on HRQoL in this population.

Few studies addressing VD supplementation on HRQol in patients with diabetes have been conducted. Krul-Poel et al. performed a randomized, double-blind, placebo-controlled trial with 275 patients with type 2 diabetes mellitus (T2DM), who monthly supplemented 50,000 IU of cholecalciferol during six months, and found no changes in QoL at the end of the study [8]. Similarly, Mager et al. analyzed the impact of administering different doses of vitamin D3 (2000 IU day or 40,000 IU monthly) for six months in T2DM patients with chronic kidney disease (CKD), and did not detect improvement in their HRQoL [9]. Those results differ from ours since the patients in the present trial received higher doses of cholecalciferol. Furthermore, both studies used cholecalciferol monthly, in contrast to cholecalciferol daily doses in our trial which have a different biological effect [12, 13, 23].

Additionally, the improvement of HRQoL in our study was found by analysis of EQ-5D-5L index after supplementation of cholecalciferol for 12 weeks. We noticed a change in the final EQ-5D-5L index using sets of values from all countries available. It was important to unify the 5 dimensions of problems in a single index to obtain our results. It is our knowledge that additional studies are necessary to establish the importance of each dimension for the Brazilian population. For that, are required both a traditional method validation, performed in Brazil in 2015, and a populational study, which has not happened in this country yet [24]. However, our findings showed improvement of EQ-5D-5L index using sets of values from countries of different continents, with different populations and the most diverse HDIs, which gives a lot of consistency to our results.

When we analysed each dimension of EQ-5D in this trial, we found an improvement of mobility. Moreover, self-care dimension showed a tendency to be better by the end of VD administration. Those results differ from Raymakers et al., who describe, in a cross-sectional study, a greater impact of anxiety/depression dimension in HRQoL, evaluating 473 patients with T1DM in Ireland [25]. Nonetheless, it is important to notice that in BrazDiab1SG, as described by Felício et al., patients in North and Northeast regions reported lower frequency of anxiety and depression when compared to other brazilian regions [3, 25]. In addition, it is known that geographic aspects, especially temperature and sun exposure, can affect quality of life [26]. The consistent improvement of EQ-5D-5L index when evaluated by sets of values from countries that represent almost all geographical regions validates our findings.

It is well known that skeletal muscle fibers carry VD receptors, and their activation is a possible mechanism of muscle growth [23, 27]. Biopsy and consistent findings of muscle atrophy were associated with VD deficiency [28]. Moreover, Sato et al. (2005) showed that calcium and vitamin D supplementation improves muscle fibers in number and size, as well as the lower members function. Randomized controlled trials and meta-analyses have shown that 25(OH)D supplementation decreases risk of falls and fractures in elderly patients [29–32]. Finally, VD deficiency is associated with muscle atrophy, musculoskeletal pain and worse physical function in the general population [32]. In our sample, most patients had hypovitaminosis D (VD < 30 ng/ml) at the beginning of the study and, by the end of the supplementation, 94% of them had sufficient levels of 25 (OH) D. It could have potentiated extra-skeletal actions of VD [33]. Thus, the hypothesis that VD affected mobility and QoL in our study by its direct action on muscle fibers must be considered.

Our results also demonstrated a reduction in albumin excretion at the end of cholecalciferol supplementation. It particularly occurred in the subgroups that improved QoL in post-hoc analysis. In addition, there was a correlation between final 25(OH)D levels and final albuminuria. Felício et al. in a pilot study that analyzed patients with T1DM who received 4,000 and 10,000 units of cholecalciferol for 12 weeks according to their previous 25(OH)D levels found a reduction in prevalence of diabetic kidney disease (DKD) and a correlation between the percentage of VD levels variation and albuminuria after cholecalciferol supplementation [11]. There is growing evidence that vitamin D given in high doses can be renoprotective [34, 35]. Humalda et al. (2015) performed a systematic review of all randomized clinical trials with calcitriol or paricalcitol as an antiproteinuric intervention. During follow-up, VD active analogs reduced proteinuria by an average of 16%. These results were obtained, in most cases (84%), in patients under chronic treatment of previous conditions with renin angiotensin aldosterone inhibitors, accentuating the capacity of VD analogs to reduce residual proteinuria. Those authors suggest that compensatory increase in renin is a paradoxical event secondary to the use of SARS inhibitors, and can be antagonized by calcitriol [36]. Furthermore, Wolfgram et al. (2017) presented a secondary analysis of the cross-sectional study SPRINT (Systolic Blood Pressure Intervention Trial), in which they evaluated a subgroup of individuals with age greater than 75 years. In addition to renal indexes (GFR and albuminuria), participants in this subgroup underwent gait speed assessment, self-assessment of HRQoL with a focus on functional status (EQ-5D), and assessment risk of falling. In the unadjusted model, the decrease in eGFR and increase in categories of albuminuria were associated with worse scores in the three tools. This data raises the hypothesis that VD may also contributes to an improvement in quality of life through its positive effect in DKD [37].

Studies have shown that, among patients with CKD, HRQoL gradually declines as the disease progresses, with the worst HRQoL scores obtained when an advanced stage of kidney disease is reached [38, 39]. When CKD coexists with diabetes an accentuated deterioration in HRQoL is expected [40]. However, few studies have examined this relationship in the early stages of CKD or attempted to incorporate albuminuria in this assessment. According to Bowling et al., albuminuria may be more sensitive than glomerular filtration rate (GFR) to identify patients with risk of functional impairment, at least in early stages of CKD [41]. In our study, abnormal creatinine levels were an exclusion criteria. Therefore, our data reinforce these findings.

The main limitations of our study were short duration (12 weeks) and absence of a placebo control group. Following this study, controlled, double-blind, randomized trials with a placebo group and a longer duration of supplementation should be performed to confirm our findings.

## Conclusions

Our data demonstrated an improvement in HRQoL after supplementation of high doses of cholecalciferol in patients with T1DM. There was an improvement in the EuroQoL utility index (EQ-5D-5L), and in mobility dimension. Additionally, the reduction of albuminuria seems to be an important factor in this context.

## Data Availability

The datasets analyzed during the current study are available from the corresponding author on reasonable request.

## Abbreviations

ANOVA: Analysis of variance test
BMI: Body Mass Index
CKD: Chronic kidney disease
DKD: Diabetic kidney disease
DM: Diabetes mellitus
GFR: Glomerular filtration rate
HDI: Human development index
HDL-C: High-density lipoprotein cholesterol
HRQoL: Health-related quality of life
HUJBB: University Hospital João de Barros Barreto
IU: International units
LDL-C: Low-density lipoprotein cholesterol
NDS: Neuropathy disability score
QoL: Quality of life
SES: Socioeconomic status
TSS: Total symptoms score
T1DM: Type 1 diabetes mellitus
T2DM: Type 2 diabetes mellitus
VD: Vitamin D
